# Role of Food Neophobia and Allergen Content in Food Choices for a Polish Cohort of Young Women

**DOI:** 10.3390/nu11112622

**Published:** 2019-11-01

**Authors:** Dominika Guzek, Joanna Pęska, Dominika Głąbska

**Affiliations:** 1 Department of Organization and Consumption Economics, Faculty of Human Nutrition and Consumer Sciences, Warsaw University of Life Sciences (SGGW–WULS), 159C Nowoursynowska Street, 02-787 Warsaw, Poland; joanna_peska@sggw.pl; 2 Department of Dietetics, Faculty of Human Nutrition and Consumer Sciences, Warsaw University of Life Sciences (SGGW–WULS), 159C Nowoursynowska Street, 02-787 Warsaw, Poland; dominika_glabska@sggw.pl

**Keywords:** Food Neophobia Scale (FNS), food neophobia, consumer choice, choice experiment, allergen, menu

## Abstract

Young women are vulnerable to a number of factors which influence their food choices, including beliefs about food products, or information about nutritional value, while information, that product is free from specific component generates consumer perceptions of its healthfulness. Among the factors which may influence such perception, there is food neophobia (FN). The aim of this study was to determine the influence of FN and information about allergens on the food product choices in the Polish cohort of young women, in the choice experiment when given a model restaurant menu. The web-based choice experiment, in a group of 600 women, aged 18–30 years, with no food allergies diagnosed, was conducted using a mock Italian-style restaurant menu. For 2 starters, 2 soups, 3 main courses and 3 desserts that were included, the allergen content, neophobic potential and perceived lack of healthiness, for a Polish population, were defined. Each respondent randomly received the version containing only a description of dishes, or a description accompanied by the allergens listed. The FN was assessed using the Food Neophobia Scale (FNS). The type of menu (with or without allergens listed) did not influence the choices of dishes. The highest FN level was observed for the women being inhabitants of villages (median of 32). The respondents characterized by a high level of FN less commonly chose dishes characterized by neophobic potential as a starter (Carpaccio), main course (Risotto ai frutti di mare) and dessert (Zabaglione). At the same time, the highest FN level was observed for respondents who chose dishes with no neophobic potential (median of 34.5). However, for allergen content and perceived lack of healthiness, no association with FN was observed, so it may be stated that for neophobic respondents, only neophobic potential is a factor limiting the choice of dishes. It may be concluded that food neophobia in young women may limit the consumption of dishes with unknown food products, and the influence is observed independently of other features of a dish, such as allergen content or perceived healthiness. The problem may appear especially for inhabitants of villages, who are characterized by the highest level of FN.

## 1. Introduction

The food choices of male and female individuals differ. This variance is due to sex-related differences in their preferences and brain responses to food products [1]. Women, especially young ones, are more vulnerable to factors other than hunger or preferences, as was observed by Wardle et al. [2] in a cohort aged 17–30, from 23 countries. Information acquired about the nutritional value of various food products, their dieting, and their beliefs about food products also influence their food choices [2]. These factors may have an important influence on their nutritional behaviors [3]. The factors gain more importance as women, compared with men, have a higher frequency of dieting [4], or even disordered eating habits [5]. But it is women who are commonly responsible for the nutrition of their whole families [6], even though their own diet is often not properly balanced [2]. This state is mainly true for young women. The situation was emphasized by a consensus statement of the International Summit on the Nutrition of Adolescent Girls and Young Women to be one requiring a high level of international priority [7]. To this end, it must be highlighted that knowledge about the food choices determinants may be necessary to conduct the dedicated education needed to influence young women’s nutritional behaviors [8].

Younger individuals are believed to be characterized by a higher sensory-specific satiety than older ones. Sensory-specific satiety is defined as the reduction of the pleasantness of a specific food product as it is consumed [9] that may promote the individual to consume a variety of products [10]. However, at the same time, young women are very prone to restrained eating [11], including weight control [12], even if they are already malnourished [13]. While combining this predilection with common nutritional beliefs, which are not always true [14], as well as with the growing popularity of a number of diets which include the elimination of specific food products, even with no medical reasons to do so [15], it may generate a problem in this group. Among popular diets, which are commonly applied even if not justified, are gluten-free diets [16] or wheat elimination diets [17], which may have a negative impact on general health if there is no medical need to apply such diet [18].

Currently, it has been observed that products with declared information on their packaging that they are free from specific components generate consumer perceptions of their healthfulness [19], even if sometimes it is just a misconception, rather than an objective assessment of the products nutritional value [20]. In this context, it should be emphasized that in spite of the fact that there are sex-dependent differences in food allergy development [21], resulting in a higher frequency of food-related symptoms of allergies and intolerances in women than men [22], more women than men follow the allergen-free diet, but a number of women following such diet even have no adverse reaction diagnosed by physicians [22]. In the study by Zysk et al. [23] on a group of women following the gluten-free diet with no justified medical reason, another factor was also observed, namely, quite a high level of food neophobia, which may cause reluctance in the individual to try new food products and as a result, cause a lower variety of consumed food products [24]. Taking this factor into account, it is also important to understand the influence of allergen content, and information about it, as well as of the food neophobia on the food product choices; hence, the aim of the present study was to determine the effect of these factors in a Polish cohort of young women in the choice experiment when given a model restaurant menu.

## 2. Materials and Methods

### 2.1. Ethics Approval Statement

The study was conducted according to the guidelines laid down in the Declaration of Helsinki. The study was approved by the Ethics Committee of the Faculty of Human Nutrition and Consumer Sciences of the Warsaw University of Life Sciences WULS-SGGW (No. 20/2017; 19.06.2017). All the participants provided their informed consent to participate in the study.

### 2.2. Study Participants

The study was conducted in a group of young Polish women that were recruited via social media using the CAWI (Computer-Assisted Web Interview) method to participate in a web-based choice experiment. The inclusion criteria were as follows: Caucasian individuals, women, age 18–30 years and declared to have no food allergy diagnosed by a physician. The exclusion criteria were as follows: not providing informed consent to participate and any missing data in the Food Neophobia Scale (FNS) questionnaire. There were no additional exclusion criteria associated with socio-economic factors, this was in order to include a wide range of participants being representative of the Polish young women. 

The participants were asked about their age (open-ended question), place of residence (closed-ended question with the following options to choose: village, town or city of <500,000 inhabitants, and city of >500,000 inhabitants), and economic status (closed-ended question with the following options to choose: very bad, bad, average, good, very good; while for the analysis very bad and bad, as well as good and very good options were combined). It was decided to ask respondents about their subjective economic status, as it was proven for Caucasians as significantly related to household income [25].

### 2.3. Choice Experiment

The web-based choice experiment was conducted using a mock Italian-style restaurant menu. Such a type of restaurant was chosen as they are very popular in Poland. In the annual research conducted in a representative sample of adult Polish respondents, Italian cuisine is always preferred by more than half of Polish consumers, as was stated also in the recent study in 2018 [26]. In some years, this type of cuisine was even graded higher than national Polish cuisine, as was observed in 2017 [27]. This predilection has resulted in a growing number of Italian and Italian-style restaurants [28].

The mock Italian-style menu was developed by a professional dietitian educated also in the field of gastronomy with practice in restaurants, including those serving Italian-style dishes. Afterwards, the menu was verified by 2 other dietitians, as well as reviewed, discussed and corrected, if needed, for its understandability and to obtain dishes being within the defined categories (for allergen content, neophobic potential and perceived lack of healthiness, as indicated in Table 1). The menu was not branded with any specific name of a restaurant, or its logotype, and was planned to be as simple as possible. The only presented information were the offered dishes in 4 categories (starters, soups, main courses, desserts) with additional information about the ingredients and recipe, including allergens, if needed. The menu was developed in two identical versions: presenting names of dishes with information about the ingredients and recipe (menu A) and presenting names of dishes with information about the ingredients and recipe, as well as for each dish, as an additional information, the allergens listed, based on Regulation (EU) No 1169/2011 [29] (menu B). For the developed menu, no prices were presented and no other information about visual appearance of dishes were provided (no photographs of dishes and no other additional information were included).

While developing the menu, an excessive number of dishes were not planned—for each course, at least two but not more than three dishes were proposed (2 starters, 2 soups, 3 main courses and 3 desserts) and no beverages were included. For each course, the dishes were characterized by a comparable energy value. At the same time, for each course, it was planned to have diverse dishes in terms of the following:

− Allergen content—defined as a content of ingredients that are among 14 major food allergens listed in Regulation (EU) No 1169/2011 [29]—it was planned to have in each category at least one dish with the noticeable allergen (as one of the main components of the dish) and at least one dish with no allergens at all.
− Neophobic potential—defined as a content of ingredients that for the Polish consumers may cause a neophobic response, due to the fact that they are not commonly consumed and are not typical of the Polish diet (the following were included: raw meat [30], seafood [31], spinach and fennel [32], and egg desserts [33] —these items are, in general, rejected by neophobic individuals)—it was planned to have in each category at least one dish with the noticeable component that may be perceived as unknown/not consumed (as one of the main components of dish) and at least one dish with no such components at all (all components neutral).
− Perceived lack of healthiness—defined as a content of ingredients that for the Polish consumers may be perceived as not healthy (the following were included: meat [34], bread [35], pasta [36], and alcohol [37] — these items are perceived by some consumers as not healthy)—it was planned to have in each category at least one dish with the noticeable component that may be perceived as unhealthy (as one of the main components of dish) and at least one dish with no such components at all (all components neutral).

The dishes and descriptions provided in a developed mock Italian-style menu for both menu A and menu B are presented in Table 1, with the defined categories of dishes for allergen content, neophobic potential and perceived lack of healthiness based on the ingredients, that were not presented in menus, except for the allergens, based on Regulation (EU) No 1169/2011 [29] that were listed for menu B.

As for the planned dishes, there were three variables included (allergen content, neophobic potential, and perceived lack of healthiness) and each of them had two options (dish defined as within criterion or not). There were 8 possibilities (2 × 2 × 2) to define a dish within the criteria. Taking it into account, it was planned to have at least one dish for one possibility (being a setup of variables). Moreover, if more than one dish was planned within the same setup of variables, they were to be dishes for various courses. The planned dishes distributed for the included variables are presented in Table 2.

The obtained mock Italian-style restaurant menu was applied in the web-based choice experiment. Each participant saw only one version of the menu (menu A with no allergens listed or menu B with allergens listed). For each individual, the system randomly chose the version and participants did not receive any information that there was more than one possible version. Each respondent was asked a simple question about her choice of dishes—she was asked to indicate the dishes that she would order while eating in this restaurant. The respondents were informed that they may choose whatever they want and they were asked to imagine a situation when they may afford whatever they want (the prices were not presented). Moreover, they were allowed to not choose a dish within each course, as they were informed that they should indicate only those dishes that they would order to consume (it was assumed that for some young women, a four-courses meal may be too large to consume).

### 2.4. Assessment of Food Neophobia

To measure the level of food neophobia, the Food Neophobia Scale (FNS) by Pliner and Hobden [38] was applied. The FNS is a 10-item scale with each item rated using a 7-point Likert scale (from 1 = strongly disagree, to 7 = strongly agree). For the presented study, the obtained answers for each question were attributed to a number of points (from 1 to 7). For 5 positive items, they were reversed for scoring and for 5 negative ones, they were not reversed. The resultant FNS ranged from 10 to 70. For the analyzed respondents, the internal consistency was assessed using Cronbach’s alpha (0.79). The result revealed good internal consistency, as it was above 0.7 [39].

While interpreting the obtained results of the FNS, the obtained scores were analyzed as a continuous variable (obtained score) and in categories (of low, medium or high levels of food neophobia). In order to stratify respondents into categories of FNS, their results were grouped according to the group mean and standard deviation (SD) values, as is commonly applied [40,41,42,43]:

− Low food neophobia level—FNS score < mean value minus SD value.
− Average food neophobia level—FNS score within the range from mean value minus SD to mean value plus SD.
− High food neophobia level—FNS score > mean value plus SD value.

### 2.5. Statistical Analysis

To verify the normality of distribution, the Shapiro–Wilk test was used. For the internal consistency testing conducted for the FNS, Cronbach’s alpha coefficient was applied. 

Three stages of analysis of data were applied, as follows:

− The influence of the information with the allergens listed on the consumer choices of dishes was verified (menu A versus menu B)—as no differences were stated, the results obtained for menu A and menu B may have been combined.
− The influence of food neophobia (FNS score) on the consumer choices of dishes was verified—as the influence was stated, a deepened analysis was conducted taking into account the general characteristics of the chosen dishes.
− The influence of food neophobia (FNS score) on the characteristics of chosen dishes—for this analysis, in terms of allergen content, neophobic potential and the perceived lack of healthiness, the choices of each respondent for each course (Table 2) were stratified into the following groups:
  (a) Choice of dishes with no allergen content; choice of 1–3 dishes with allergen content; choice of all dishes (4 of them) with allergen content.
  (b) Choice of dishes with no neophobic potential; choice of 1–3 dishes with neophobic potential; choice of all dishes (4 of them) with neophobic potential.
  (c) Choice of dishes with no perceived lack of healthiness; choice of 1–3 dishes with perceived lack of healthiness; choice of perceived lack of healthiness (4 of them) with perceived lack of healthiness.

To compare the frequency in sub-groups, the chi-squared test was used. To identify the differences between groups, due to the non-parametric distributions, the U Mann–Whitney test was used (for 2 groups) or Kruskal–Wallis Analysis of Variance (ANOVA) with multiple comparisons of results (for more than 2 groups). The *p* ≤ 0.05 was accepted as the level of significance. Statistical analysis was performed using Statistica software version 8.0 (StatSoft Inc., Tulsa, OK, USA).

## 3. Results

### 3.1. Characteristics of the Studied Group 

The basic characteristics of the studied group of 600 women is presented in Table 3. The majority of recruited participants (aged 18–30) were inhabitants of big cities (over 60%) and assessed their economic situation as either good or very good (55%).

The FNS scores in the studied group of women are presented in Table 4. It was observed that the median score in the group was 29, while for the sub-groups of low, average and high food neophobia level they were 16 (*n* = 104), 30 (*n* = 407) and 44 (*n* = 89), respectively.

The FNS scores in the studied group of women stratified by place of residence and economic status are presented in Table 5. It was observed that the place of residence influenced the observed FNS score, as the highest food neophobia level was observed for the women being inhabitants of villages (*p* = 0.0290).

### 3.2. Influence of the Information with the Allergens Listed on the Consumer Choices 

The comparison of the choices of dishes stratified by information about allergen content (type of menu) is presented in Table 6. It was observed that for none of the courses did the type of menu influence the choices of dishes, as the frequency of choosing a specific dish within a course was similar, independently from the received menu (*p* > 0.05).

### 3.3. Influence of the Food Neophobia on the Consumer Choices 

As it was observed that the information about allergen content (type of menu) does not influence the choices of dish, the respondents were analyzed combined, to assess the influence of the FNS score.

The choice of dishes compared between groups stratified by the FNS scores of respondents is presented in Table 7. It was observed, that for starters, main courses and desserts, the FNS scores influenced choices. The respondents characterized by a high level of food neophobia less commonly chose Carpaccio as a starter (*p* = 0.0058), Risotto ai frutti di mare as a main course (*p* < 0.0001) and Zabaglione as a dessert (*p* < 0.0001). All of them were defined as dishes characterized by neophobic potential, while for Carpaccio and Zabaglione, perceived lack of healthiness was also defined and for Risotto ai frutti di mare and Zabaglione, allergen content was also indicated.

**Figure 1:**
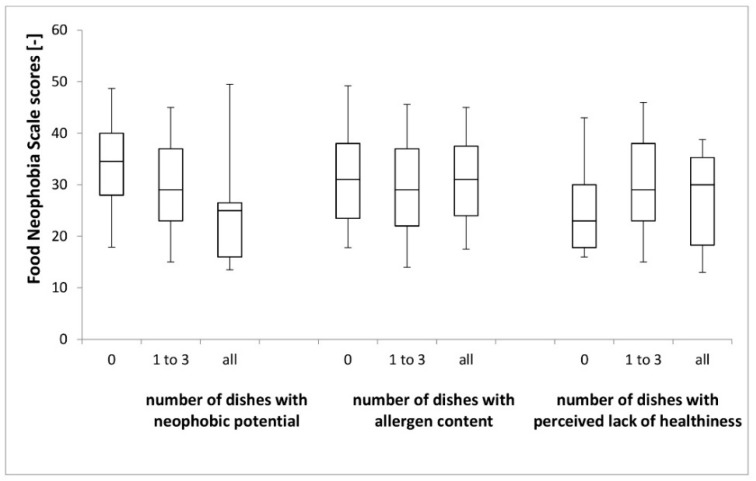
Food Neophobia Scale (FNS) scores of respondents compared between groups stratified by choice of dishes in the categories of neophobic potential, allergen content and perceived lack of healthiness.

The FNS scores of respondents compared between groups stratified by choice of dishes in the categories of neophobic potential, allergen content and perceived lack of healthiness are presented in Figure 1. 

The highest FNS scores (highest food neophobia level) were noted for respondents who chose dishes with no neophobic potential (*p* = 0.0051), confirming the previous analysis. However, while comparing participants who chose 1–3 dishes with neophobic potential and all of them with neophobic potential, it was stated that there were no differences between them. At the same time, for allergen content (*p* = 0.8443) and perceived lack of healthiness (*p* = 0.1206), there were no significant differences.

## 4. Discussion

Many popular diets are restricted ones that focus on avoiding some food products or their components, which results in misleading information being promoted by the media that some products, e.g., gluten, lactose, fat, are harmful always and for everybody. A survey by Watson [44] found that 65% of American adults think that gluten-free products are healthier than regular ones, and over 27% choose gluten-free products to lose weight. This situation is driven by several factors, not only media messages but also consumer-directed marketing strategies by manufacturers and press reports of the clinical benefits of gluten avoidance influence people [45]. As a result, it is observed that food products labeled as ‘free from’ any ingredient can be perceived as better for health, in comparison with products with no such information [19]. However, following a restricted diet, based on avoiding some type of products, could result in a deficiency in healthy individuals without health issues, which was indicated as a possible health risk in a national Polish study in a representative cohort of girls and young women [46]. For example, gluten-free processed grain products are often lower in fiber, iron, zinc, and potassium than normal grain products [47]. Even if the consumers believe that this trend of restricted diets is healthy and that they have an optimal diet, they commonly do not meet the requirements of the dietary guidelines [48].

Taking all these factors into account, in the present study, the influence of information about allergen content was assessed. It was analyzed to determine if this information had any impact on the consumer choice. For the applied mock Italian-style restaurant menu, women receiving menus with allergens listed made comparable choices to women receiving the menus with no allergens listed. This may be interpreted as a lack of influence of allergen content on the choices of dishes in the assessed group of young women, but it cannot be stated for a fact that this is the general approach in the studied group. The similarity in choices may be attributed to the growing trend of so-called cheat-meals, being not in accordance with the applied general diets, which are often consumed while being in restaurants, or eating out [49]. At the same time, such lack of differences for the applied menus with and without allergens listed may result from the fact that for a person avoiding specific food products or components, the name of the product may be sufficient information to indicate that it may be a source of a specific allergen, as it is observed that for consumers with and without food allergies, the level of knowledge about allergens is similar [50]. It is also associated with the fact that consumers avoiding some allergens, who are not able to identify products with allergen and without, would commonly rather avoid those allergens and products from the same group as these are perceived as suspicious products. This was observed for tree nut allergic individuals [50]. 

The additional fact that may be indicated here is a general lack of trust in food allergen labels declared by consumers [51]. Moreover, they report that some ingredient lists include too much information, as well as too-technical terms, so they are hard for them to understand [52]. As a result, consumers often declare that they are not satisfied with the current allergen labelling practices, which results in their personal stress and feelings of insecurity [53].

While analyzing the indicated approaches, it may be supposed, that the studied women with no food allergies diagnosed may not avoid any allergens from their diet, but based on literature, this is a rare situation. For example, in the United States of America, it was observed that 30% of inhabitants are interested in avoiding gluten [16]. At the same time, it may be supposed that in the studied group, respondents either do not avoid allergens in a restrictive way and while eating out, they do not follow their individual restriction, or that they preventively avoid some products which they suspect to contain allergens, even based on the limited information. Both approaches for individuals with no food allergies cannot be interpreted as improper, if only they were not associated with a decreased quality of diet due to overconsumption in social situations [54], excessive food products avoiding that may result in reduced variety of food products consumed [55].

The other factor influencing consumer food choices is food neophobia which was, in the present study, the major determinant of choice of dishes from the applied mock Italian-style restaurant menu. It was stated that women defined as neophobic more than others chose dishes without components of neophobic potential, and that women choosing dishes without components of neophobic potential have the higher FNS scores. However, it was not associated with allergen content and perceived healthiness of dish, so this may indicate, that for neophobic ones, specifically neophobic potential of food products was a determinant of choice, but not other factors. Taking this into account, it must be indicated that in spite of the fact that food neophobia is a rejection of novel or unknown food products that occurs mainly in children [56] and should decrease with age [57], for adults, it also significantly determines food choices. This is in agreement with the stable level of food neophobia from adolescence (approximately 13 years old) through adulthood suggested by some researchers [58]. At the same time, it should be emphasized that Tuorila et al. [40] observed that food neophobia may increase in older people, so food neophobia may not be so stable for adults as was previously supposed.

As a food neophobia may influence dietary habits, it may result in lower quality of diet, inadequate dietary intake [59] and consequent deficiencies [60]. Some studies indicated that food neophobia was related to reduced vegetable intake [42,60], or vegetable and fruit intake [60,61], while it was more strongly associated with vegetable than with fruit intake [59]. However, so far, there is no study published that analyzes the influence of food neophobia on choice of dishes from the restaurant menu. The study of Camarena et al. [62] analyzed the influence of ethnocentrism and food neophobia on the ethnic food consumption in Spain, but for the Polish population, Italian cuisine, which is analyzed in the present study, should not be considered as an ethnic one. This type of cuisine was introduced in Poland in the XVI century and is nowadays quite popular in all the regions of the country [26]. Hence, the reason for rejecting some dishes by neophobic respondents must have been associated with a low level of familiarity with those specific dishes (novel food) or with the specific ingredients (unknown food products). 

As was mentioned previously, some ingredients may cause a neophobic response due to the fact that they are not commonly consumed and are not typical of the Polish diet such as raw meat [30], seafood [31], spinach and fennel [32], and egg desserts [33]. Therefore, the dishes that included those ingredients may be rejected by neophobic individuals. Such a situation can be easily explained by analyzing the basic mechanisms; however, individual differences must also be taken into account. 

The reduced acceptance of meat and fish products was stated in several studies. An evolutionary explanation was presented in that the reluctance to use these products was due to a protective mechanism against the possibility of accidental poisoning by raw or perishable food products [30,33]. It is in general indicated that animal food products are a more likely source of food poisoning than non-animal foods, so this mechanism may be also attributed to seafood and eggs [33]. At the same time, in the case of the vegetables, the bitter taste may be rejected due to the natural subconscious mechanism of defense of the organism from poisoning, as most potential poisons in nature have a bitter taste [63]. Taking all these factors into account, the dishes defined as those of neophobic potential may be justified and their avoidance by neophobic respondents may be explained. 

However, avoidance of some products by respondents with food neophobia may lead to a reduced variety of products consumed. The resultant reduced quality of the respondent’s diets accompanied by possible inadequate dietary intake of some nutrients may lead to some deficiencies. In the analyzed group, such risk may be observed especially for respondents from villages, as the place of residence influences consumers to a higher food neophobia level. At the same time, the economic status was not a determinant of food neophobia in the studied group, which allows to conclude that women from villages are especially prone to have a reduced quality of diet due to their food neophobia.

In spite of the fact that the presented study revealed interesting new observations from a large homogenic sample of respondents, associated with the influence of food neophobia in young women, which may limit the consumption of dishes with unknown food products, some limitations of the study should be also listed. The study was conducted only in a group of young women, so the situation in other groups is still unknown, and it was conducted only using one mock Italian-style menu, so the observations should be verified for other menus, including not only Italian-style, but also the other ones. Moreover, the influence of broad interfering factors should also be included in further studies.

## 5. Conclusions

It may be concluded that food neophobia in young women may limit the consumption of dishes with unknown food products, and the influence is observed independently of other features of the dish, such as allergen content or perceived healthiness. It may cause not properly balanced diets, due to avoiding some foods and a reduced diversity of consumed products. The problem may appear greater especially for inhabitants of villages, who are characterized by the highest level of food neophobia. This problem should be taken into account in the education programs and public health policy.

## Figures and Tables

**Table 1:** The dishes and descriptions provided in a developed mock Italian-style menu, as well as defined categories of dishes for allergen content, neophobic potential and perceived lack of healthiness based on the ingredients.

Dishes and Descriptions Provided in a Mock Italian-Style Menu			Defined Categories of Dishes Based on the Ingredients		
Course	Dish	Provided Description	Content of Ingredients that are Among 14 Major Food Allergens Listed in Regulation (EU) No 1169/2011 [29]	Content of Ingredients that for the Consumers may Cause the Neophobic Response	Content of Ingredients that for the Consumers may be Perceived as not Healthy
Starter	Carpaccio	Paper-thin raw beef slices	–	Raw meat	Meat
	Caprese	Slices of tomatoes and mozzarella	Milk and products thereof (including lactose) (annex 2, pt. 7)	–	–
Soup	Minestrone	Vegetable soup with zucchini, green beans, carrot, spinach and fennel	–	Spinach and fennel	–
	Pappa al pomodoro	Tomato bread soup	Cereals containing gluten (annex 2, pt. 1)	–	Bread
Main course	Risotto ai frutti di mare	Seafood risotto with parmesan	Crustaceans and products thereof (annex 2, pt. 2); milk and products thereof (including lactose) (annex 2, pt. 7); molluscs and products thereof (annex 2, pt. 14);	Seafood	*–*
	Spaghetti alla siciliana	Spaghetti with tomatoes and roasted eggplant	Cereals containing gluten (annex 2, pt. 1)	–	Pasta
	Bistecca alla fiorentina	Beef steak served with rosemary roasted potatoes	–	–	Meat
Dessert	Tiramisu	Espresso-dipped ladyfingers with mascarpone-egg cream	Cereals containing gluten (annex 2, pt. 1); eggs and products thereof (including lactose) (annex 2, pt. 3); milk and products thereof (including lactose) (annex 2, pt. 7)	Egg cream	–
	Zabaglione	Egg yolk dessert with alcohol	Eggs and products thereof (including lactose) (annex 2, pt. 3)	Egg dessert	Alcohol
	Granita	Frozen fruit dessert	–	–	–

**Table 2:** The planned dishes distributed for the included variables (allergen content, neophobic potential and perceived lack of healthiness).

Variables			Planned Dish	Course
Allergen Content	Neophobic Potential	Perceived Lack of Healthiness		
−	−	−	Granita	Dessert
+	−	−	Caprese	Starter
−	+	−	Minestrone	Soup
−	−	+	Bistecca alla fiorentina	Main course
+	+	+	Zabaglione	Dessert
+	+	−	Risotto ai frutti di mare	Main course
			Tiramisu	Dessert
+	−	+	Pappa al pomodoro	Soup
			Spaghetti alla siciliana	Main course
−	+	+	Carpaccio	Starter

**Table 3:** Basic characteristics of the studied group of women (*n* = 600).

Characteristics		Results
Age (years)	Mean ± SD	21.2 ± 1.9
	Median (range)	21 * (18–30)
Place of residence	Village	72 (12.0%)
	Towns and cities <500,000 inhabitants	153 (25.5%)
	Cities >500,000 inhabitants	375 (62.5%)
Economic status	Very bad and bad	36 (6.0%)
	Average	234 (39.0%)
	Good and very good	330 (55.0%)

* non-parametric distribution (verified using Shapiro–Wilk test: *p* ≤ 0.05). SD: Standard deviation.

**Table 4:** Food Neophobia Scale (FNS) scores in the studied group of women (*n* = 600).

FNS Score	Total (*n* = 600)	Food Neophobia Level		
		Low ^1^ (*n* = 104)	Average ^2^ (*n* = 407)	High ^3^ (*n* = 89)
Mean ± SD	30.2 ± 10.1	16.1 ± 2.4	30.3 ± 5.7	46.3 ± 5.9
95% CI	29.4–31.0	15.6–16.5	29.8–30.9	45.1–47.5
Median	29 *	16 *	30 *	44 *
Range	10–70	10–19	20–40	41–70
25th–75th	23–37	14–18	26–35	42–49

* non-parametric distribution (verified using Shapiro–Wilk test: *p* ≤ 0.05); ^1^ low level (< mean value − SD); ^2^ average level (the range from mean value − SD to mean value + SD); ^3^ high level (> mean value + SD) [40,41,42,43]. CI: Confidence intervals.

**Table 5:** Food Neophobia Scale (FNS) scores in the studied group of women (*n* = 600) stratified by place of residence and economic status.

Characteristics		Mean ± SD	Median (range)	*P*-Value
Place of residence	Village	33.5 ± 9.9	32 (15–56) ^a^	0.0070
	Towns and cities <500,000 inhabitants	30.2 ± 9.6	29 (10–56) ^b^	
	Cities >500,000 inhabitants	29.6 ± 10.2	29 * (11–70) ^b^	
Economic status	Good and very good	29.9 ± 10.4	29 *(10–70)	0.1775
	Average	31.0 ± 9.6	31 (11–58)	
	Very bad and bad	28.2 ± 10.3	30 (13–50)	

* non-parametric distribution (verified using Shapiro–Wilk test: *p* ≤ 0.05); ^a,b^ different letters indicate significant differences between groups (*p* < 0.05).

**Table 6:** Comparison of the choices of dishes stratified by information about allergen content (type of menu).

Course	Dish	Total	Menu A ^1^ (*n* = 300)	Menu B ^2^ (*n* = 300)	*p*-Value
Starters	Carpaccio	193 (34.8)	97 (35.3)	96 (34.4)	0.8302
	Caprese	361 (65.2)	178 (64.7)	183 (65.6)	
Soup	Minestrone	178 (33.9)	92 (35.0)	86 (32.8)	0.6671
	Pappa al pomodoro	347 (66.1)	171 (65.0)	176 (67.2)	
Main course	Risotto ai frutti di mare	149 (24.2)	70 (21.8)	79 (23.5)	0.4499
	Spaghetti alla siciliana	281 (45.8)	131 (40.8)	150 (44.7)	0.1408
	Bistecca alla fiorentina	184 (30.0)	120 (37.4)	107 (31.8)	0.3125
Dessert	Tiramisu	377 (55.1)	182 (53.8)	195 (56.4)	0.3106
	Zabaglione	90 (13.2)	39 (11.5)	51 (14.7)	0.2085
	Granita	217 (31.7)	117 (34.6)	100 (28.9)	0.1740

^1^ menu A—standard menu with information about the ingredients and recipe; ^2^ menu B—standard menu with information about the ingredients and recipe followed by allergens listed.

**Table 7:** Choice of dishes compared between groups stratified by Food Neophobia Scale (FNS) scores of respondents.

Course	Dish	Total	Level of Food Neophobia			*p*-Value
			Low ^1^	Average ^2^	High ^3^	
Starters	Carpaccio	193 (34.8)	49 (48.0)	123 (32.7)	21 (27.6)	0.0058
	Caprese	361 (65.2)	53 (52.0)	253 (67.3)	55 (72.4)	
Soup	Minestrone	178 (33.9)	27 (31.0)	125 (34.9)	26 (32.5)	0.7581
	Pappa al pomodoro	347 (66.1)	60 (69.0)	233 (65.1)	54 (67.5)	
Main course	Risotto ai frutti di mare	149 (22.7)	49 (40.8)	92 (20.6)	8 (8.9)	<0.0001
	Spaghetti alla siciliana	281 (42.7)	36 (30.0)	200 (44.7)	45 (50.0)	
	Bistecca alla fiorentina	227 (34.6)	35 (29.2)	155 (34.7)	37 (41.1)	
Dessert	Tiramisu	377 (55.1)	61 (51.7)	264 (57.0)	52 (50.5)	<0.0001
	Zabaglione	90 (13.2)	19 (16.1)	63 (13.6)	8 (7.8)	
	Granita	217 (31.7)	38 (32.2)	136 (29.4)	43 (41.7)	

^1^ low level (< mean value − SD); ^2^ average level (the range from mean value − SD to mean value + SD); ^3^ high level (> mean value + SD) [40,41,42,43].
